# DNA Repair in Nucleosomes: Insights from Histone Modifications and Mutants

**DOI:** 10.3390/ijms25084393

**Published:** 2024-04-16

**Authors:** Kathiresan Selvam, John J. Wyrick, Michael A. Parra

**Affiliations:** 1 School of Molecular Biosciences, Washington State University, Pullman, WA 99164, USA; 2 Department of Chemistry, Susquehanna University, Selinsgrove, PA 17870, USA

**Keywords:** histone post-translational modifications, nucleotide excision repair, base excision repair, histone methylation, oncohistones

## Abstract

DNA repair pathways play a critical role in genome stability, but in eukaryotic cells, they must operate to repair DNA lesions in the compact and tangled environment of chromatin. Previous studies have shown that the packaging of DNA into nucleosomes, which form the basic building block of chromatin, has a profound impact on DNA repair. In this review, we discuss the principles and mechanisms governing DNA repair in chromatin. We focus on the role of histone post-translational modifications (PTMs) in repair, as well as the molecular mechanisms by which histone mutants affect cellular sensitivity to DNA damage agents and repair activity in chromatin. Importantly, these mechanisms are thought to significantly impact somatic mutation rates in human cancers and potentially contribute to carcinogenesis and other human diseases. For example, a number of the histone mutants studied primarily in yeast have been identified as candidate oncohistone mutations in different cancers. This review highlights these connections and discusses the potential importance of DNA repair in chromatin to human health.

## 1. Introduction

Cellular DNA is continually disfigured by endogenous and exogenous DNA damaging agents, including reactive oxygen species (ROS), alkylating agents, ultraviolet (UV) radiation, and a myriad of other causes of DNA lesions [1]. Such damage must be efficiently recognized and repaired by cellular repair enzymes in order to prevent the accumulation of mutagenic and cytotoxic DNA lesions. The activities of these repair enzymes are typically coordinated in one or more single- or multi-step repair pathways. These pathways include (1) nucleotide excision repair (NER), which recognizes and excises bulky, helix-distorting DNA lesions [2,3,4,5]; (2) base excision repair (BER), which removes specific classes of small DNA base lesions [6,7,8]; (3) mismatch repair (MMR), which removes DNA mismatches typically arising during replication [9,10]; (4) direct damage reversal, such as the light-dependent repair of UV lesion by photolyase enzymes or repair of O6-methyl-guanine alkylation lesions by the O6-methylguanine DNA methyltransferase suicide enzymes [11,12]; (5) single-strand break repair, which repairs single-stranded DNA gaps [13]; (6) double-strand break (DSB) repair, consisting of homologous recombination (HR), nonhomologous end joining (NHEJ), and other alternative end joining pathways [14,15]; and (7) post replication repair (PRR), which includes translesion DNA synthesis (TLS) by specialized TLS polymerases opposite replication-stalling DNA lesions [16].

In eukaryotic cells, all of these repair pathways must function in the context of a DNA template packaged with histone proteins into nucleosomes [17,18]. Individual nucleosomes are comprised of ~147 bp of DNA wrapped nearly two times around an octamer of histone proteins [19,20,21]. This octamer typically contains two copies each of histones H2A, H2B, H3, and H4. Each of these histone proteins has the same basic core structure, consisting of three alpha helices and two loops, which comprise the histone-fold domain [22]. The individual histones differ substantially in their N-terminal and C-terminal extensions from the core histone-fold domain, which are largely unstructured, but in some cases contain additional secondary structure elements, including an additional C-terminal alpha helix (αC) in histone H2B and an N-terminal helix (αN) in histone H3 [21,22]. The central organizing unit of the histone octamer is a heterotetramer consisting of H3 and H4 proteins. To the central H3/H4 heterotetramer are bound two H2A/H2B heterodimers, each binding to opposite sides or ‘faces’ of the heterotetramer [22].

**Figure 1:**
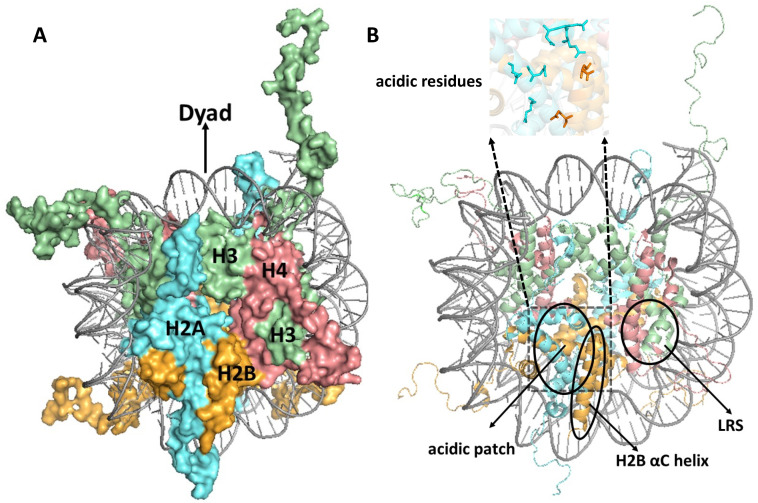
(**A**) Structure of the nucleosome. The different histones are indicated as follows: H3, H4, H2A, and H2B are shown in pale green, salmon red, cyan aquamarine, and bright orange, respectively. The central dyad axis is indicated at the top of the nucleosome. (**B**) Nucleosome structure highlighting unique structural features, including the acidic patch, the histone H2B αC helix, and loss of ribosomal silencing (LRS) surface. Figures were generated using pymol from PDB ID:1KX5.

The nucleosomal DNA is sharply bent as it binds to this histone octamer, with the protein–DNA interactions primarily confined to 14 superhelical locations (SHL) where the minor groove of the DNA is oriented toward the histone octamer (i.e., SHL-6.5 to SHL6.5) [19,20,21]. These interactions primarily occur between the DNA backbone and structural features of the core histone-fold domains (i.e., L1/L2 loops or a1/a1 helices), with the exception of the most distal DNA exit/entry points (SHL ± 6.5), which interact with residues in the H3 αN helix [21]. If the central dyad axis of the nucleosomal DNA is oriented upward, then the H3–H4 heterotetramer generally binds DNA near the top half of the nucleosome, while the H2A/H2B dimers bind DNA near the bottom of the nucleosome (Figure 1A). The N-terminal tails of each histone exit either between the two DNA gyres (i.e., histones H2B and H3) or to the side of one DNA gyre (i.e., histones H2A and H4), extending a considerable length beyond the nucleosome core (Figure 1A). The C-terminal tail of H2A traverses the nucleosome face and exits near the dyad axis at the top of the nucleosome, while the C-terminal tail of H2B forms a unique feature on the nucleosomes surface (see below).

A nucleosome can be thought of as resembling the wheel of a car, with the DNA being the ‘tire’ that is bound to the lateral surface of the histone octamer, which can be thought of as the ‘rim’ of the wheel (Figure 1A). Using this analogy, the nucleosome ‘face’ or disk surface can be thought of as the side or face of the wheel, containing the spokes and hub. The nucleosome face contains a number of unique features that are often targeted by proteins that interact with nucleosomes. These include (1) the acidic patch, comprised of a number of aspartic acid, glutamic acid, and other H2A/H2B residues which form a deep groove on the nucleosomes surface (Figure 1B) that is targeted by many nucleosome-binding proteins [22,23,24]; (2) the H2B αC helix, which forms a ridge that runs from the center to the bottom of the nucleosome face (Figure 1B) and is ubiquitylated at H2B K123 in yeast or K120 in mammalian cells [22,25]; and (3) the loss of ribosomal silencing (LRS) surface (Figure 1B), consisting of H3/H4 residues that surround the methylated H3 lysine-79 (H3K79) residue [22,26]. Since most DNA in a eukaryotic genome is packaged in nucleosomes, proteins involved in DNA transcription, replication, and repair are thought to exploit these different nucleosome features in order to specifically bind to nucleosomes (as opposed to free histones) and promote these critical DNA metabolic processes in nucleosomal DNA.

## 2. Impact of Nucleosomes on Genome-Wide DNA Damage, Repair, and Mutagenesis

The wrapping of DNA around the histone octamer to form a nucleosome has profound consequences to DNA damage, repair, and mutagenesis, particularly in tumor genomes. In many cancers, somatic mutations are elevated in nucleosomal DNA relative to flanking linker DNA or nucleosome-free regions [27,28,29]. This pattern can be potentially explained by both biochemical studies and genome-wide repair experiments, indicating that the repair of many DNA lesions is inhibited inside nucleosomes [17,18,27,28,29,30,31,32,33,34,35,36,37,38]. While many cancers showed elevated mutation frequencies in nucleosomes, a few, namely, lung squamous cell carcinoma and lung adenocarcinoma, show the opposite trend with elevated mutations in linker DNA [28]. However, the molecular mechanism responsible for elevated mutation rates in nucleosome-free linker DNA in lung cancers remains an open question.

Recent studies indicate that mutation patterns in cancers are also modulated by the positioning of the minor groove of the DNA helix relative to the histone octamer [27,28,29]. Again, many cancers showed elevated mutation frequencies at positions in the nucleosomal DNA where the minor groove faces the histone octamer (i.e., ‘minor-in’ rotational settings). This can be potentially explained by biochemical and genome-wide studies indicating that BER is particularly inhibited at minor-in positions [17,18,28,32,34,39,40]. In contrast, in certain cancers (e.g., lung cancers and melanoma), somatic mutations are elevated at minor-out rotational settings [28,29], where the minor groove of the nucleosomal DNA faces away from the histone octamer. In the case of skin cancers, such as melanoma, elevated mutation rates at minor-out rotational settings can largely be attributed to elevated levels of UV damage formation at these positions in nucleosomal DNA [28,29,33,41,42]. We and others have found that this nucleosome ‘photofootprint’ is likely caused by DNA bending into the major groove at minor-out positions in nucleosomes [43,44], resulting in a DNA structure more susceptible to forming UV photoproducts such as cyclobutane pyrimidine dimers (CPDs).

While many of the studies cited above concern inhibition of DNA excision repair pathways (i.e., NER and BER) by nucleosomes, the packaging of DNA into nucleosomes also impacts other repair pathways. For example, biochemical studies indicate that nucleosomes inhibit repair by the MMR pathway [45], consistent with bioinformatic analysis indicating that inhibition of MMR in nucleosomes may contribute to elevated somatic mutation rates, particularly at minor-in rotational settings, in esophageal cancers [46]. As another example, DNA resection during DSB repair by the HR pathway almost certainly requires nucleosome repositioning and/or eviction [14,47].

Finally, while the packaging of DNA into nucleosomes can inhibit many repair processes, nucleosomes also serve as an essential platform to promote DNA damage signaling (see below). Moreover, many cellular enzymes, including ATP-dependent nucleosome remodeling enzymes and histone modifying enzymes (e.g., histone acetyltransferases, methyltransferases, ubiquitin ligases, etc.) can promote efficient repair of DNA lesions in nucleosomes, as detailed below.

## 3. Roles of Histone Post-Translational Modifications in Repair

Histone residues are decorated with a variety of post-translational modifications (PTMs) that act in a coordinated and ordered manner to facilitate dynamic changes in chromatin for the tight regulation of cellular processes such as DNA replication, transcription, damage response, and repair [48,49,50]. In recent years, there has been remarkable progress in identification of histone modifications, characterizing their genome-wide distribution and understanding their function in regulating transcription, replication, and repair.

The first key theme that has emerged from these studies Is that many of these histone PTMs have dual functions in distinct cellular processes, such as transcription and repair. For example, acetylation of lysine residues located in the flexible histone N-terminal tails by lysine (K) acetyltransferases (KATs) play a critical role in regulating transcription [48,51,52]. In yeast, Gcn5 and Esa1, which serve as the catalytic subunits of the SAGA [53,54] and NuA4 [55] KAT complexes, respectively, regulate the transcription of many yeast genes by acetylating N-terminal lysine residues in histones H2B and H3 (Gcn5) and H2A and H4 (Esa1) [56]. Histone acetylation by these same KATs also promotes efficient NER of UV damage in yeast [57,58,59,60,61] and, in the case of Gcn5, in human cells [62,63]. Histone acetylation is thought to promote transcription in part by recruiting ATP-dependent chromatin remodeling (ACR) complexes, such as SWI/SNF and RSC in yeast [64,65]. This recruitment is mediated by bromodomains in the subunits of these ACR complexes, as bromodomains specifically bind acetylated lysine residues (e.g., [64,65,66,67]). These same ACR complexes are also required for efficient NER in yeast [68,69,70]; to what extent their recruitment and activity during repair is mediated by histone acetylation remains unclear.

**Figure 2:**
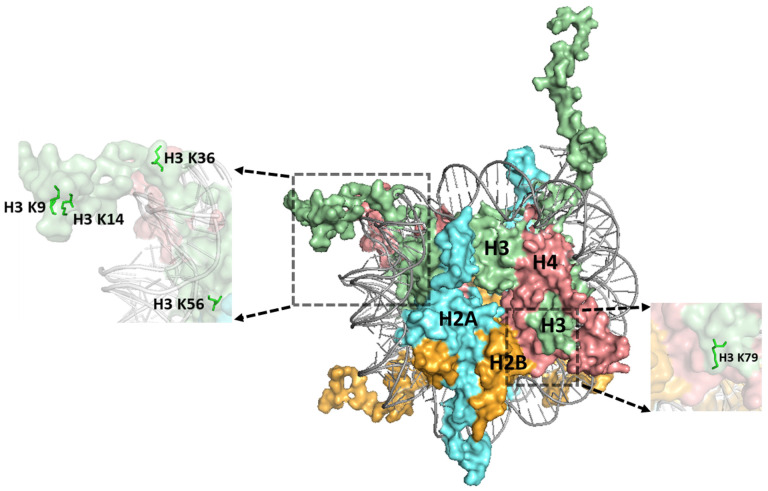
Structure of the nucleosome highlighting a number of histone residues whose post-translational modifications (PTMs) are implicated in repair. Histone H3 K9, H3 K36, and H3 K79 are methylated and histone residues H3 K9, H3 K14, and H3 K56 are acetylated. Histones are indicated as follows: H3, H4, H2A, and H2B are depicted in pale green, salmon red, cyan aquamarine, and bright orange, respectively. The figure was generated using pymol from PDB ID:1KX5.

Lysine residues in the structured histone core domain are also acetylated, a primary example being H3 lysine-56 (K56), which is located in the αN helix of histone H3 (Figure 2). H3 K56 is acetylated during the process of histone deposition during replication, and therefore plays overlapping roles in nucleosome assembly, DNA damage response, and checkpoint recovery [71,72,73,74,75,76]. An in vitro study indicates that H3 K56 acetylation may also limit some forms of DNA replication, in this case by DNA polymerase beta, which performs error-prone DNA repair synthesis during BER [77,78]. H3 K56 acetylation has also been reported to promote histone and tRNA gene transcription [79,80,81], as well as NER [82], indicating that this PTM, which directly modulates nucleosomal DNA unwrapping [83,84,85], also multitasks in promoting different cellular functions in chromatin.

Histone lysine residues are also methylated by lysine methyltransferases (KMTs) [48,86], which play a clear role in establishing and maintaining different chromatin states in the genome. A second theme from recent studies is that such pre-existing chromatin states profoundly impact the repair of many different classes of DNA lesions. For example, H3 K9 methylation (Figure 2) is important for the establishment and maintenance of heterochromatic regions [86], and such regions of the genome have elevated somatic mutation rates in many cancers, including skin cancer [87]. Elevated somatic mutation rates can be explained by other studies indicating that such heterochromatic regions are refractory to repair [27,35,88]. H3 K9 methylation also plays critical roles in silencing repeat sequences, including different classes of mobile DNA elements, and regulating tissue-specific gene expression during cellular differentiation [86]. Since genomic regions marked by H3 K9 methylation are typically silenced, and are therefore dispensable for cellular function and homeostasis, repair inhibition in such regions might redirect the limiting cellular repair machinery to instead prioritize fixing damage in more important (and accessible) regions of the genome.

In contrast, trimethylated H3 K36 (H3 K36me3) is enriched at transcribed exon sequences [89,90,91]. Like H3 K9 methylation, H3 K36 methylation (Figure 2) multitasks to perform a number of important cellular functions, including regulating RNA splicing [92,93,94,95] and repressing the initiation of cryptic transcription [92,96,97,98,99]. H3 K36 methylation also plays an important role in regulating DNA repair. For example, a key MMR protein known as MSH6 contains a PWWP domain that specifically binds H3 K36me3, thereby targeting the MMR machinery to preferentially fix replication errors in transcribed exons [100,101,102,103]. Hence, the MMR machinery prioritizes the repair of critical protein-coding exons by recognizing and exploiting pre-existing methylation marks associated with this chromatin state.

Pre-existing histone PTMs have been reported to affect repair even at the level of individual nucleosomes. A genome-wide study of repair of DNA alkylation damage induced by methyl methanesulfonate (MMS) exposure in yeast revealed that pre-existing histone PTMs modulate BER in nucleosomes [32]. This study found that nucleosomes marked by high levels of pre-existing histone acetylation (e.g., H3 K14 acetylation, H3 K23 acetylation, etc.) display more rapid BER of DNA alkylation damage at more distal locations in nucleosomes (i.e., near DNA entry/exit sites in nucleosomes) but paradoxically slower repair of damage near the central nucleosome dyad. Analysis of MMS-induced mutations in yeast revealed a similar pattern, with elevated mutation density in H3 K14 acetylated nucleosomes near the slower-repairing nucleosome dyad [32]. In contrast, nucleosomes marked with H3 K36 methylation show the opposite pattern of BER in nucleosomes, and MMS-induced mutations show a similar trend [32].

A third key theme is that DNA dam”ge a’d its subsequent repair often changes the epigenetic landscape of histone PTMs. Perhaps the best studied example is the phosphorylation of serine-139 residue in the C-terminal tail of the histone variant H2AX adjacent to DNA DSBs, a histone PTM called γH2AX [104,105,106]. Phosphorylation of H2AX by DNA damage signaling kinases such as ATM in human cells, which can extend to nucleosomes as far as a megabase from the DSB, plays an important role in repair [105,107].

Histone ubiquitination, which consists of the covalent attachment of a ubiquitin protein to a target lysine residue by the concerted action of E2 ubiquitin conjugating enzyme and E3 ubiquitin ligase, also plays a critical role in DSB signaling and repair [108,109,110]. At DSBs, the human E3 ubiquitin ligases RNF20/40 mono-ubiquitinate H2B at lysine-120 [111,112,113], while RNF168 E3 ligase catalyzes ubiquitination of H2A (or H2AX) lysine-13 and lysine-15 (K13/K15) [114,115,116,117], in order to promote DNA damage signaling and DSB repair. Similar histone PTM alterations occur at DSBs in yeast, although the canonical yeast histone H2A (which resembles human H2AX) is phosphorylated at serine-129 (S129) and the yeast Bre1 E3 ubiquitin ligase, the homolog of human RNF20/RNF40 dimer complex, ubiquitinates yeast H2B K123 to promote DSB signaling and repair [118,119].

Histone ubiquitination is also altered during NER. The CUL4-DDB-ROC1 E3 ubiquitin ligase complex acts to ubiquitinate lysine residues in histones H3 and H4 in response to UV damage, thereby facilitating the recruitment of a key NER damage sensor known as XPC [120]. On the contrary, H2B K123 is deubiquitinated in yeast in response to RNA polymerase II stalling at UV lesions [121]. Notably, damage-dependent H2B deubiquitination, which promotes efficient NER, is catalyzed in part by the ubiquitin-specific protease Ubp8, a subunit of the Gcn5-containing SAGA complex. Gcn5 has also been reported to stimulate histone H3 acetylation in response to UV damage [60], which promotes efficient NER in chromatin [58,59,122,123]. Hence, two different enzymatic activities in the SAGA complex are thought to alter the histone PTM landscape in order to facilitate repair of UV damage. Thus, chromatin alterations associated with the initial steps of NER (e.g., damage recognition, etc.) can alter the landscape of histone PTMs. Furthermore, the later NER step of re-synthesizing the excised DNA strand can also alter histone PTMs [124,125].

Finally, single-stranded DNA breaks and other DNA lesions trigger the recruitment and activation of poly(ADP-ribose) polymerase 1 (PARP1), which catalyzes extensive poly(ADP)ribosylation (PARylation) of PARP1 itself or other target proteins, including histones [48,126]. PARylation promotes the recruitment of XRCC1 and its associated repair proteins, such as LIG3 (DNA Ligase 3), to facilitate single-strand break repair [127,128,129,130,131]. While PARP1 plays a critical role in single-strand break repair and BER, PARP1 also facilitates chromatin remodeling during NER, via the ACR ALC1, through its interactions with the NER damage sensor XPC [132]. Notably, PARP1 activity can significantly deplete cellular stores of key metabolic cofactor NAD^+^, leading to significant perturbations in cellular metabolism, as well as pathology in individuals with genetic defects in these repair pathways [133,134].

## 4. Histone Mutants That Affect DNA Repair

Mutants in histone proteins in yeast have been extensively utilized to characterize the role of specific histone domains, residues, and PTMs in different cellular functions, including transcription, replication, chromatin assembly, and DNA repair [49,135,136,137,138,139,140,141,142,143,144,145,146,147,148,149,150,151,152]. These efforts culminated in a trio of reports generating and characterizing libraries of histone mutants in yeast [153,154,155], in which essentially every histone residue was individually mutated (typically to alanine) and the resulting mutant phenotype was characterized. These studies yielded a number of surprising findings. First, relatively few of the histone alanine mutants were lethal in yeast grown in rich media. For example, in histones H2A and H2B, only four residues were essential for yeast viability in rich media, and all of these (yeast H2A Y58, E62, D91, and H2B L109) were located in the acidic patch [153,155] (Figure 1B). Second, many histone mutants showed sensitivity to DNA damaging agents, including the alkylating agent MMS and UV light [153,154,156,157]. Elucidating how these histone mutants perturb DNA damage tolerance, repair, and/or signaling remains an ongoing challenge, although common mechanisms have emerged from recent studies.

First, some histone mutants cause DNA damage sensitivity and repair defects by down-regulating the expression of one or more key DNA repair genes. For example, histone H4 K16R and K16Q mutations rendered yeast cells sensitive to UV damage and cause defects in NER [158]. These mutants were further shown to compromise UV-induced expression of a number of key NER genes (i.e., *RAD23*, *RAD4*, *RAD16*, *RAD1*, *RAD2*, and *RAD14*), which, along with changes in chromatin accessibility, can potentially explain the observed NER defects [158].

Similarly, simultaneous deletion of both the histone H2A and H3 N-terminal tails caused MMS sensitivity and a defect in BER of MMS-induced lesions in yeast [135]. This BER-defect turned out to be caused by reduced expression of the *MAG1* gene, which encodes a DNA glycosylase that plays a critical role in initiating BER of DNA alkylation damage. Overexpression of Mag1 in yeast cells lacking the H2A and H3 N-terminal tails rescued the BER defect, but did not restore MMS resistance [135]. Subsequent experiments indicated that the MMS sensitivity of the H2A and H3 tail mutants was epistatic with a mutation in the *RAD18* gene, which encodes an E3 ubiquitin ligase that functions in PRR by ubiquitinating proliferating cell nuclear antigen (PCNA). PCNA ubiquitination was compromised in the H2A and H3 tail mutants, suggesting a mechanism by which the histone H2A and H3 N-terminal tails function in PRR of MMS-induced DNA lesions [135].

**Figure 3:**
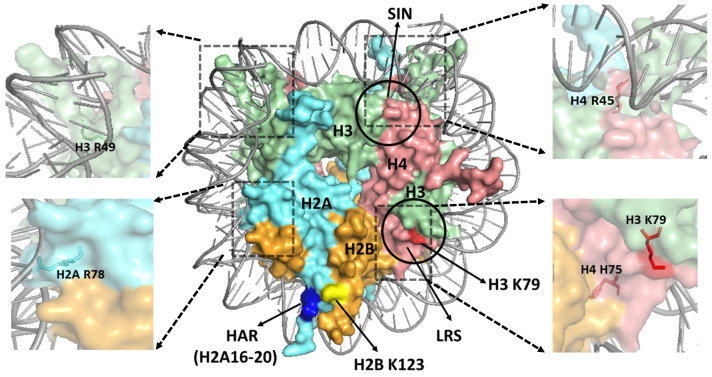
Structure of a yeast nucleosome, highlighting key regions in the nucleosome surface. The loss of ribosomal silencing (LRS) and Sin^−^ (SIN) domains are indicated with circles. The histone residues H3 K79, H2B K123, and the histone H2A repression (HAR) domain (i.e., H2A residues 16–20) are indicated in red, yellow, and blue color, respectively. The positions of the sprocket arginine residues H2A R78, H3 R49, and H4 R45 are indicated in zoomed images. The location of H3K79 residue (red) in the central region of LRS domain and the neighboring H4 H75 residue (salmon red) is also depicted in a zoomed image. The figure was generated using pymol from PDB ID:1ID3.

A second mechanism is that some histone mutants cause DNA damage sensitivity because they are sites of PTMs critical for DNA repair, DNA damage signaling, etc. For instance, the histone H3 K79E mutation, which mutates a lysine in the LRS domain of the nucleosome face (Figure 2 and Figure 3) that is methylated by the Dot1 KMT [159], renders yeast cells sensitive to UV light [160]. Deletion of the yeast *DOT1* gene also causes a similar level of UV sensitivity, and epistasis analysis indicates that Dot1 and H3 K79 function in NER and post-replication repair [160]. Subsequent studies [139,161] indicate that Dot1 and H3 K79 function to promote repair by an NER sub-pathway known as global genomic-NER (GG-NER), which directly senses and repairs helix-distorting DNA lesions throughout the genome [4]. However, the mechanism by which H3 K79 methylation by Dot1 promotes GG-NER in yeast remains unclear. DOT1L catalyzed H3 K79 methylation in mammalian cells has been shown to recruit the key GG-NER sensor XPC to UV damage sites to promote efficient NER [162]. Consequently, defects in H3 K79 methylation are associated with increased frequency of UV-induced melanoma development in mice [162], highlighting the potential importance of this pathway in preventing skin carcinogenesis.

While histone H3 K79 regulates GG-NER, the histone H3 K36A mutant in yeast affects a different NER sub-pathway known as transcription coupled-NER (TC-NER) [163], which specifically repairs DNA lesions on the transcribed strand of genes. TC-NER is triggered by RNA polymerase II stalling at a helix-distorting DNA lesion, which is detected by a key TC-NER factor known as CSB in human cells and Rad26 in yeast [2,5,164,165,166]. H3 K36A mutants in yeast show UV sensitivity that is epistatic with mutations in *RAD26*, indicating that this histone residue functions in TC-NER [163]. H3 K36 is methylated in yeast by the Set2 KMT [167], and deletion of the *SET2* gene in yeast also causes UV sensitivity that is epistatic with a *RAD26* mutant, and a genome-wide defect in TC-NER of UV damage [163]. Set2 associates with the elongating RNA polymerase II and methylates H3 K36 during transcription elongation [94,168]. We have postulated that RNA polymerase II stalling at a lesion may result in the accumulation of H3 K36 methylation behind the stalled polymerase, potentially serving as an epigenetic signal for TC-NER [163]. Consistent with this model, a previous study suggested that H3 K36 methylation may promote Rad26 recruitment [169].

While mutations in H3 K36 cause UV sensitivity in a wild-type strain background, the H3 K36A mutant paradoxically rescues the UV sensitivity of a GG-NER deficient (*rad16*∆) yeast strain [163]. Set2-catalyzed histone H3 K36 methylation functions as a signal to recruit lysine deacetylases (KDAC) enzymes, which remove acetylation marks from nucleosomes in the transcribed regions of yeast genes [97,98,99]. This pathway plays a critical role in suppressing cryptic transcription, which initiates from inside of genes and proceeds in either the sense or antisense direction [97,99,170]. Mutants in *SET2* or H3 K36 activate cryptic transcription, resulting in antisense transcription of the non-transcribed strand (NTS) of yeast genes. Normally, the NTS is only repaired by GG-NER, but in *set2∆* or H3 K36A mutants, it can also be repaired by TC-NER associated with cryptic anti-sense transcription of the NTS [163]. Hence, the partial rescue of UV sensitivity in GG-NER deficient cells is likely due to increased repair resulting from cryptic TC-NER of the NTS of these genes. As Set2 homologs in human cells (i.e., NSD1, NSD2, NSD3 and SETD2) are frequently mutated in human cancers [94,171], these findings may have important implications in carcinogenesis and chemotherapy resistance.

Histone H3 K36 mutations have also been identified in a subset of human cancers and are thought to contribute to carcinogenesis [172,173,174,175,176,177]. One of the most studied of these ‘oncohistone’ mutations is a K36M mutation that occurs in the histone H3 variant H3.3. H3 K36M mutations occur in nearly 90% of cases of a bone cancer called chondroblastoma [178,179] but have also been identified in pediatric soft tissue sarcoma, head and neck squamous cell carcinoma, melanoma, bladder, and colorectal cancer [174,180,181,182]. The H3 K36M is a dominant mutation that binds to and inhibits the activity of SETD2 and other H3 K36 methylating enzymes in human cells [178].

Oncohistone mutations in a neighboring H3 G34 residue have also been identified in human cancers and disrupt H3 K36 methylation. Two of the most common G34 mutants, namely, G34R and G34V, are observed in H3.3 [174] in cerebral cortex tumors [172]. Other variants such as G34W and G34L are found in giant cell tumors of bone [179]. The G34R/V/W variants decrease H3K36me2 and H3K36me3 methylation in *cis* (i.e., on the same histone H3), but not in *trans* like the K36M mutation [183,184]. The H3.3 G34R/V/D oncohistone mutants also block interaction with the key mismatch repair protein MSH6 and therefore display a mutator phenotype [184].

**Figure 4:**
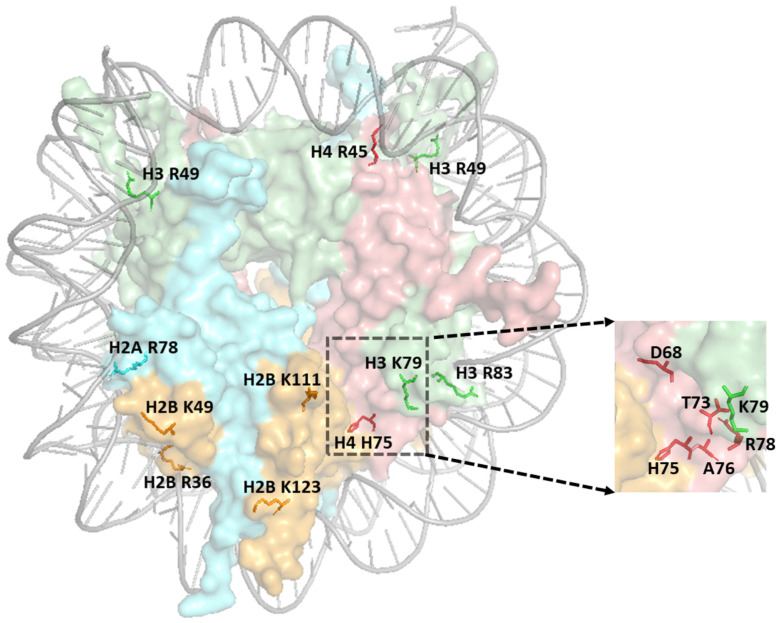
Structure of nucleosome highlighting histone residues (labeled with histone and residue number) implicated in DNA repair. The zoomed image indicates the residues within the LRS domain. The figure was generated using pymol from PDB ID:1ID3.

While the modification states of the histone residues discussed above (e.g., H3 K36 and H3 K79 methylation) have been extensively studied, proteomics studies have also detected PTMs in other histone residues, which, although obscure, appear to contribute to DNA damage repair or signaling. For example, histone H2B lysine-46 (K46) in mammalian cells was previously reported to be methylated and acetylated [136,185]. Mutation of the homologous H2B residue in yeast to alanine (i.e., H2B K49A) resulted in a UV sensitivity phenotype [136]. Similarly, H2B lysine-108 (K108) in mammalian cells has been reported to be methylated [185], and a mutation in the homologous lysine residue in yeast H2B (H2B K111, see Figure 4), which has also been reported to be methylated [186], results in MMS sensitivity [136]. Notably, subsequent proteomics studies have indicated that H2B K108 methylation is down-regulated in postmortem brain samples from Alzheimer’s disease patients [187]. However, not all histone PTM sites function in the DNA damage response and affect genome stability. For example, H2B lysine-37 (K37) was discovered to be dimethylated in yeast and potentially higher eukaryotes, but mutating this residue did not render yeast cells sensitive to DNA damaging agents [188].

A third mechanism is that some histone mutants cause DNA damage sensitivity by altering the modification state of other histone residues. For instance, a previous study indicated that a small domain (residues 16–20) comprising a ‘knuckle’ helix in the histone H2A N-terminal domain represses the expression of many yeast genes, and it was therefore labeled the histone H2A repression (HAR) domain [141,189]. Deletion of the HAR domain in yeast rendered cells UV-sensitive, although the mechanism involved was initially unclear [141]. A subsequent study indicated that the HAR domain is required for histone H2B K123 ubiquitination and H3 K4 methylation, likely because it is adjacent to H2B K123 in the nucleosome structure (Figure 3) and functions as a docking site for H2B ubiquitinating enzymes [190]. Since H2B K123 ubiquitination plays an important role in DNA repair [121,161], and H2B K123 mutants are sensitive to UV irradiation [191], the role of the HAR domain in promoting histone H2B ubiquitination can potentially explain the UV sensitivity of the HAR mutant. Surprisingly, mutations in HAR domain residues, including in residues R17 and S19 of human H2A, are observed in human cancers [174], although their potential role in carcinogenesis is unclear.

A fourth (and final) mechanism by which histone mutants can cause damage sensitivity is by affecting the structure and dynamics of the nucleosome or its binding interfaces with other proteins. One of the key structural features of the nucleosome is that a histone arginine residue inserts into the minor groove of the DNA at each minor-in rotational setting of the nucleosomal DNA, which can be compared to the teeth of a bicycle sprocket (i.e., arginine residues) inserted into the chain (DNA). Many of these ‘sprocket’ arginines have important roles in the structure and dynamics of the nucleosome and also affect DNA damage and repair [192]. For example, H4 R45 is a sprocket arginine that inserts into the DNA minor groove at SHL ± 0.5 (Figure 3), which flank the central dyad (SHL0) [21]. H4 R45C and R45H mutations were originally identified in a yeast genetic screen because they alleviate the requirement for the SWI/SNF ACR complex in transcriptional activation, likely because these mutants increase intrinsic nucleosomal DNA disassociation, mobility, and sliding [193,194], and are therefore called SWI/SNF-independent (Sin^−^) mutations [26,195]. A subsequent study indicated that histone H4 R45H or R45C mutations promote UV resistance in yeast and facilitate repair of UV damage by NER, likely by enhancing the accessibility of DNA lesions to repair proteins [196,197]. H4 R45C or R45Q mutants are also observed in human cancers and may function as oncohistone mutations [174]. Notably, other Sin^−^ mutations identified in yeast are also prevalent in human cancers, including histone H3 E105K/Q, and have been hypothesized to potentially promote carcinogenesis [174].

Other sprocket arginines also play roles in DNA damage sensitivity and repair, including H2A R78 (SHL ± 5.5) and H3 R49 (SHL ± 6.5, see Figure 3 and Figure 4). Unlike H4 R45C, which promotes UV resistance and repair, alanine mutations in either of these residues result in elevated UV sensitivity in yeast and a defect in NER of UV-induced CPD lesions [192]. Both the H2A R78A and H3 R49A mutants are also MMS sensitive, as are mutants in other histone sprocket arginine residues (i.e., H3 R83A and H2A R43A) [153,154,156,192,198]. However, none of these mutants affect BER of MMS-induced DNA alkylation damage [192], indicating that they likely affect a different repair or damage tolerance pathway.

The mechanism by which the H2A R78 and H3 R49 residues promote NER in chromatin remains unclear. The H2A R78A mutant does not affect expression of any known NER gene, indicating that it may directly affect NER activity [192]. Structural studies and biochemical experiments suggest that H2A R78 may play an important role in interacting with the H2A/H2B chaperones FACT and Nap1 [199,200,201]. It has also been reported that the H2A R77A mutation in human H2A (homologous to H2A R78 in yeast) affects the nucleosome sliding activity of an ACR in vitro [202]. These findings suggest possible mechanisms by which H2A R78 regulates NER in yeast, since both FACT and ACR complexes have been linked to promoting NER activity in chromatin [68,69,132,203,204,205,206,207,208].

One of the sprocket arginine residues that lacked any observable phenotype when mutated was H2B R36 (SHL ± 4.5, see Figure 4) [192]. This residue is located in a highly basic region of histone H2B called the histone H2B repression (HBR) domain, because it plays an important role in transcriptional repression in yeast [209]. While the H2B R36A mutant lacks any observable phenotype, deletion of the entire HBR domain (H2B ∆30–37) causes UV sensitivity in yeast, and previous studies indicate that the HBR mutant affects UV damage formation, as well as repair at specific loci [137,209]. The HBR domain is located between the two DNA gyres as the H2B tail exits the nucleosome core [25]. Biophysical studies indicate that the HBR domain plays an important role in nucleosomal DNA unwrapping and sliding and modulates the activity of BER enzymes in vitro [210]. The HBR domain also plays an important role in FACT-mediated nucleosome disassembly and assembly [211,212], which may potentially explain its role in NER.

While the discussion above focuses on sprocket arginine residues, many other histone residues also affect nucleosome stability, dynamics, and binding interfaces and potentially play a role in DNA damage sensitivity and repair. For example, a recent study used saturation mutagenesis targeted to a small region of histone H4 associated with the LRS domain to identify histone mutants that affected UV sensitivity in yeast. This study identified 24 mutations that either enhanced or reduced UV sensitivity, one of which (histone H4 H75E) significantly decreased GG-NER activity [213]. Although the H4 H75E mutant did not affect the methylation of neighboring H3 K79 (Figure 3), it impaired chromatin binding of the key DNA damage sensor Rad4. Other H4 mutants identified in the screen (e.g., H4 T73D, T73F, T73Y, R78I, D68I, A76T, R78S, and T80L, see Figure 4) affected PRR by TLS DNA polymerases, resulting in reduced UV-induced mutagenesis [214]. This study not only identified new histone mutants that affect repair and the DNA damage response, but also suggests that current histone mutant libraries, which primarily consist of substitutions to alanine, may be missing non-alanine substitutions that have important biological phenotypes. Consistent with this idea, most of the candidate oncohistone mutations identified in human cancers are not alanine substitutions [174,202]

Surprisingly, histone H4 H75 was recently discovered to be mutated (H4 H75R) in a neurological and developmental syndrome, suggesting that it plays important functions in human development [215]. The same study also found that other patients with germline H4 R45C Sin^−^ mutations displayed similar neurological and developmental symptoms [215], although the underlying molecular mechanism responsible for these phenotypes remains unclear.

## 5. Conclusions

In summary, it is clear that the packaging of DNA into nucleosomes profoundly affects both DNA damage formation and repair, and consequently contributes to altered somatic mutation rates in various cancers. Nucleosomes not only act to restrict access to the repair machinery, but can also serve as a key signaling platform in which both pre-existing and repair-associated histone PTMs can facilitate and prioritize repair of DNA lesions. The availability of libraries of histone mutants in yeast has revealed a number of key residues that regulate DNA repair pathways. It is remarkable that a number of these histone mutants characterized by genetic studies in yeast have recently been identified in human cancers and neurological disorders. It will be important in future studies to elucidate the mechanisms by which these and other histone mutants and PTMs potentially contribute to carcinogenesis and other human diseases.
